# Triplane Fracture of the Distal Femur in the Paediatric Population: A Case Report and Literature Review

**DOI:** 10.7759/cureus.7416

**Published:** 2020-03-25

**Authors:** Patrick Carroll, Niall McGoldrick, Patrick O'Toole

**Affiliations:** 1 Trauma & Orthopaedic Surgery, Cork University Hospital, Cork, IRL; 2 Paediatric Orthopaedic Surgery, Our Lady's Children's Hospital, Crumlin, Dublin, IRL

**Keywords:** orthopedics, triplane fracture, paediatric, distal femur, trauma, orthopaedics, paediatric orthopaedics

## Abstract

Triplane fractures of the distal femur in paediatric populations are extremely rare. Only three cases of paediatric triplane fractures of the distal femur are described in the literature. Our aim is to add to the scant literature on triplane fractures of the distal femur in the paediatric population. The majority of trauma and orthopaedic surgeons are not expected to come across this injury throughout their careers. A CT is recommended to plan surgery, and subsequent follow up is crucial to provide the child with the best possible outcome. In particular, follow up is important to monitor for any abnormal growth or deformities.

## Introduction

Triplane fractures of the distal femur in children are extremely rare. Fractures to the growth plate of the distal femur have been recognized as significant injuries in the literature as far back as 1894 [1]. 

The epidemiology of distal femoral growth plate injuries is well described. They represent from one to five percent of all physeal injuries, and approximately seven percent of lower extremity injuries; less than one percent of paediatric fractures [2-4]. Most are Salter-Harris type II fractures which occur in adolescents [5]. 

The fusion of the distal femoral growth plate occurs between 14 and 16 years of age for females, and 16 to 18 years of age for males [6-9]. Due to the thicker perichondral ring and periosteum, it takes more energy to injure the growth plate of a child than an adolescent [8]. 

Fractures involving the distal femur growth plate can have severe implications for children. Complications include malunion, premature closure of part or all of the growth plates with the corresponding angular and/or rotational deformities, or leg-length discrepancies, and neurovascular injuries [4].

Triplane fractures are well described of the distal tibia, distal humerus, distal radius, and the hand [10-14]. To date, only three cases have been reported of triplane fractures in the distal femur in the paediatric population [4, 6, 15]. Only one of these cases involved a female, and only one case had a pre-operative computed tomography (CT) scan performed [4, 15].

Our aim is to add to the scant literature on triplane fractures of the distal femur in the paediatric population. We describe our management and the challenges this case presented to us. In providing more information on triplane distal femoral fractures in the paediatric population, we hope to provide detailed information for surgeons who are planning to repair such fractures in the future. 

## Case presentation

**Figure 1:**
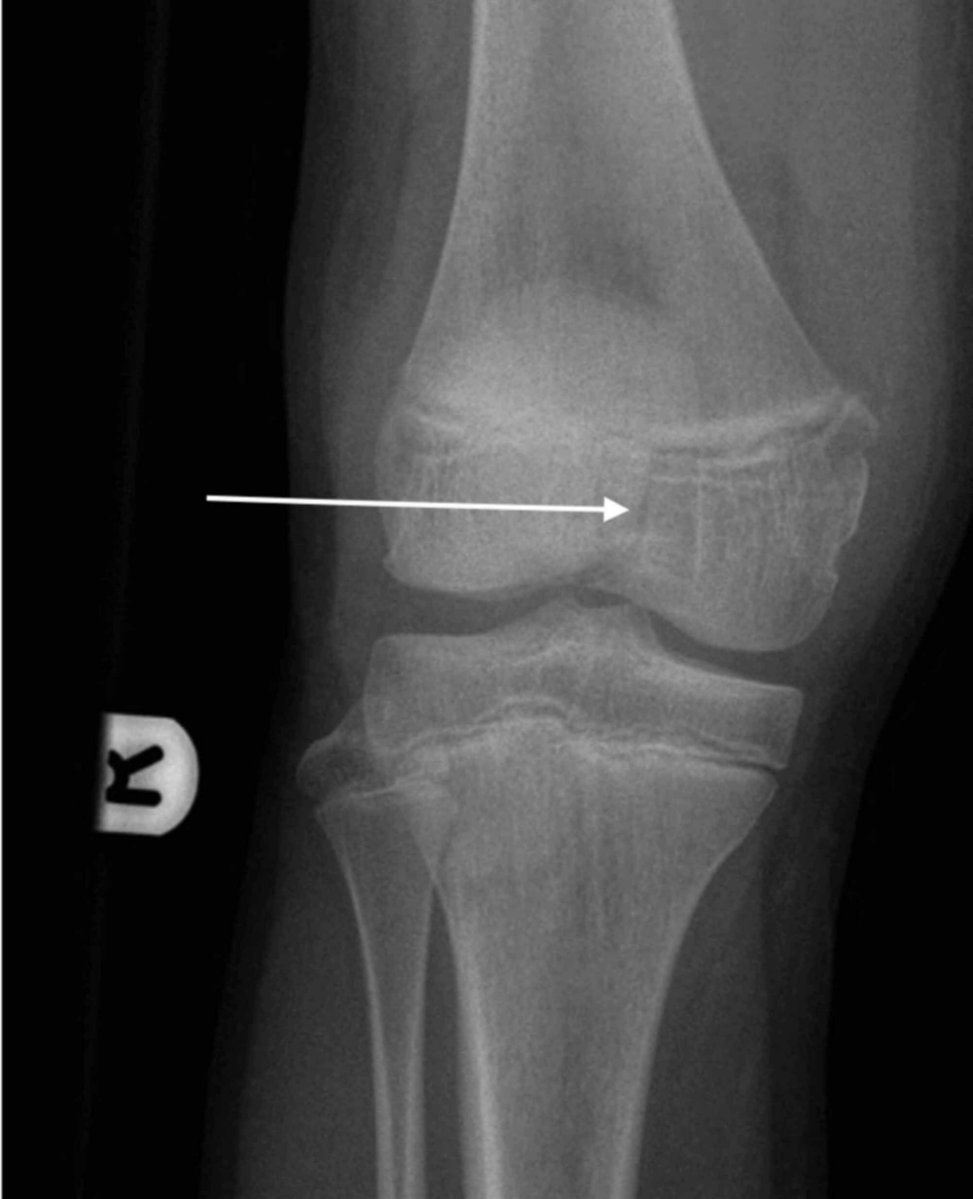
Anteroposterior x-ray of right knee post-injury

**Figure 2:**
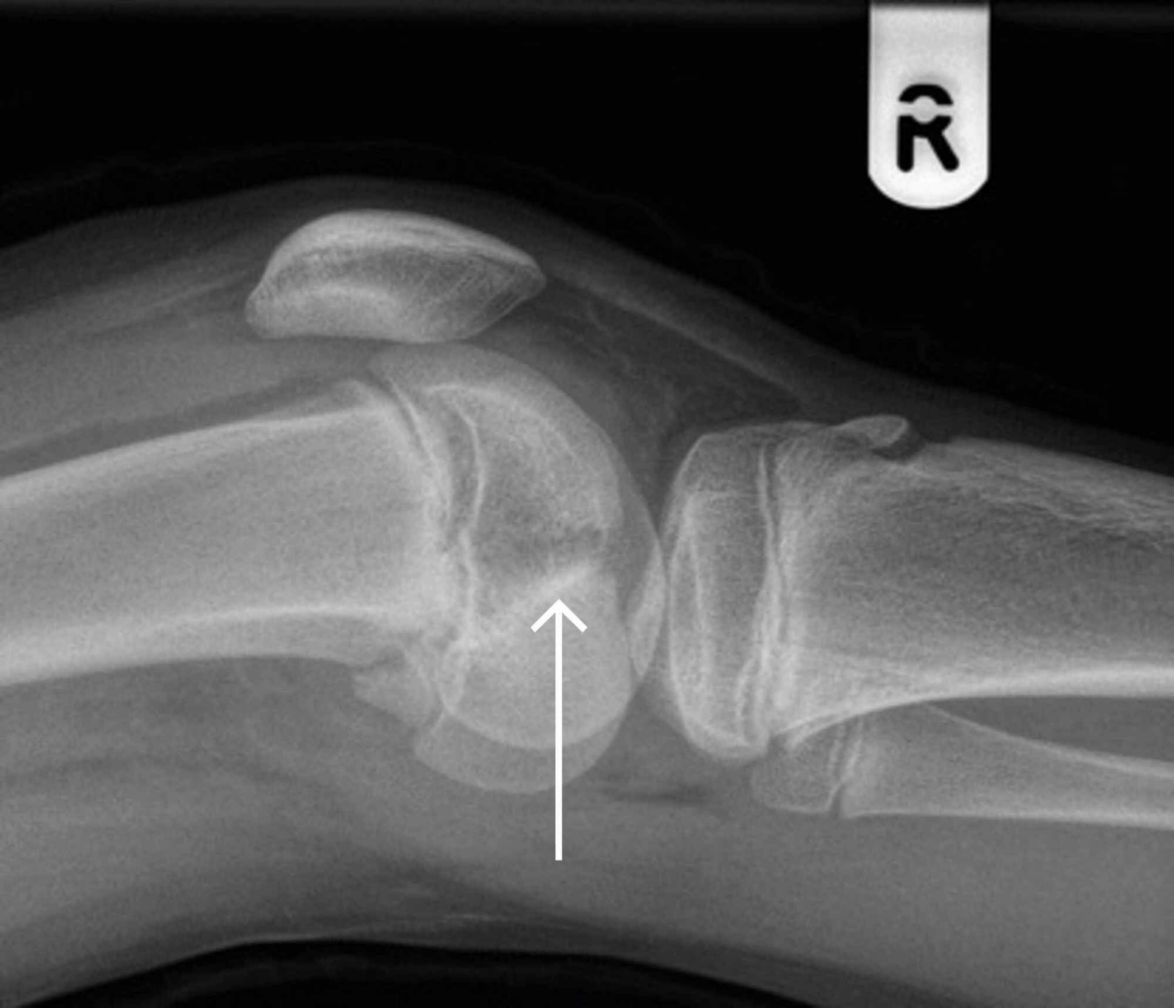
Lateral x-ray of right knee post-injury

A 13-year-old girl injured her knee while playing soccer. She tackled a goalkeeper and sustained a direct blow to the right knee. She presented to a local emergency department due to right knee pain and an inability to weight-bear. She underwent assessment by the emergency department staff, had plain film x-rays (Figures 1 and 2), and was referred to the trauma and orthopaedic surgery team on call at our institution for advice, as is the norm for paediatric fractures presenting to this local hospital. 

**Figure 3:**
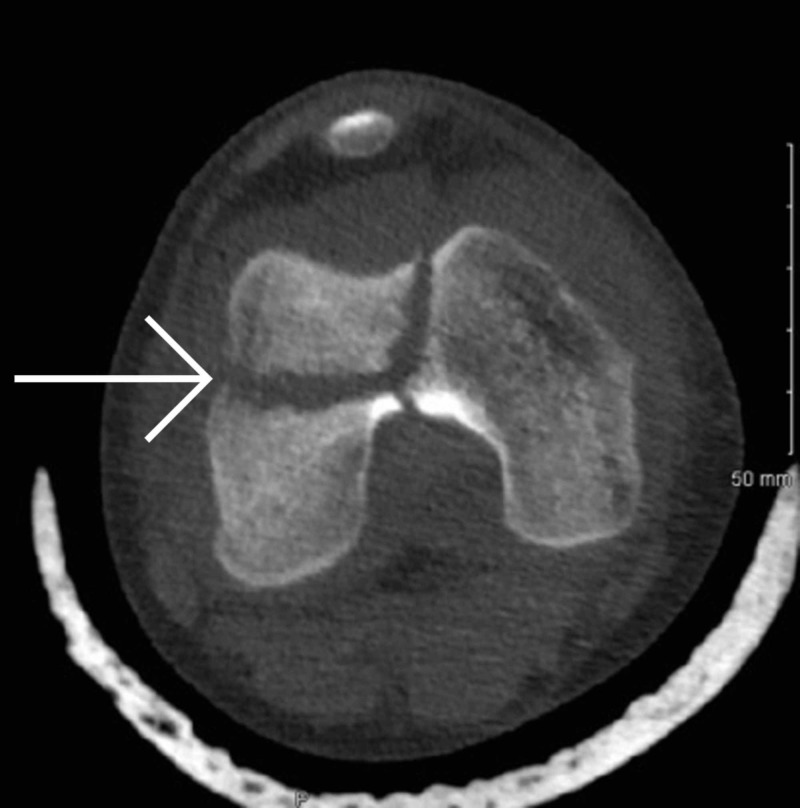
Axial view of computed tomography scan of right distal femur post-injury

**Figure 4:**
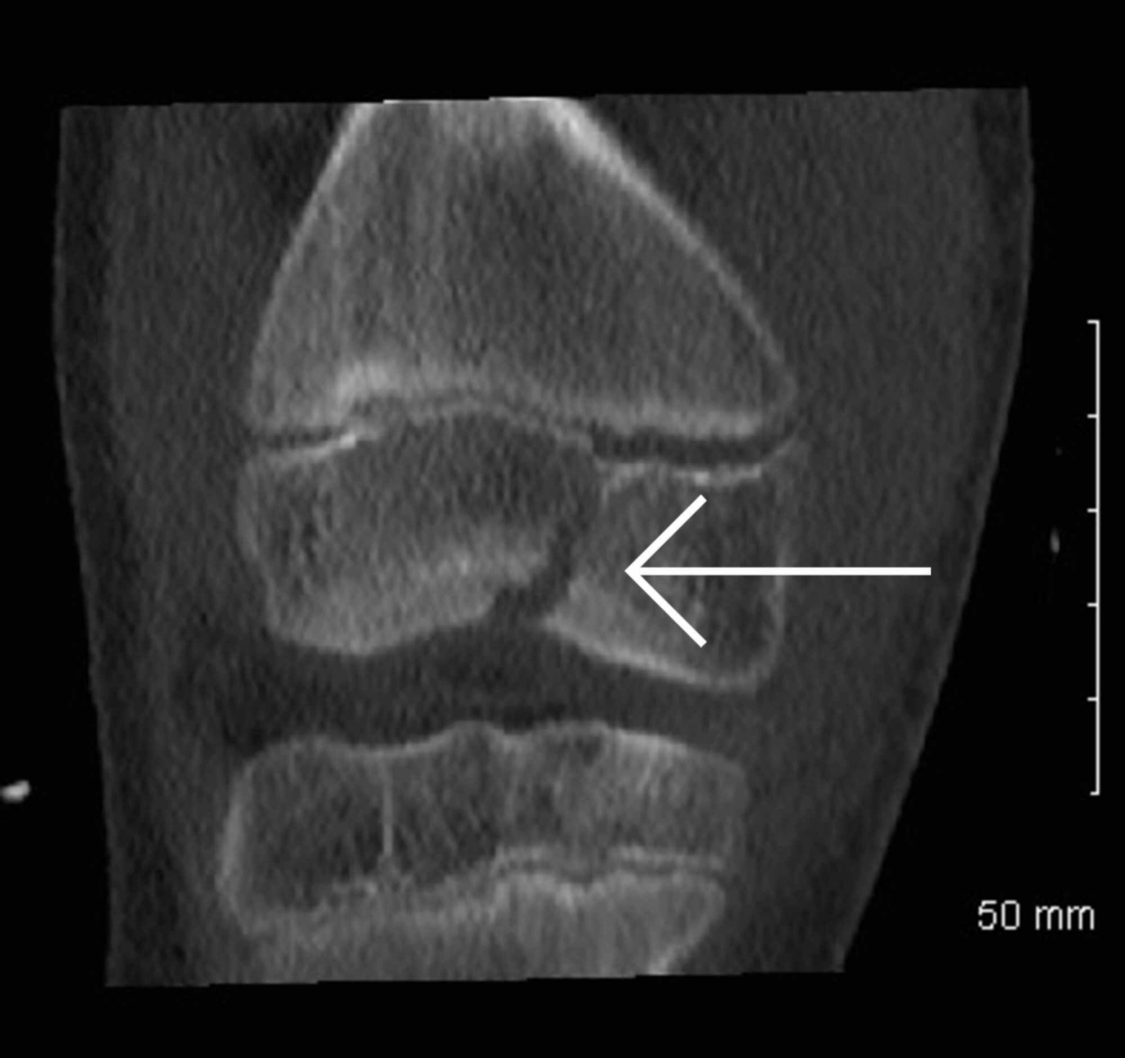
Coronal view of computed tomography scan of right knee post-injury

**Figure 5:**
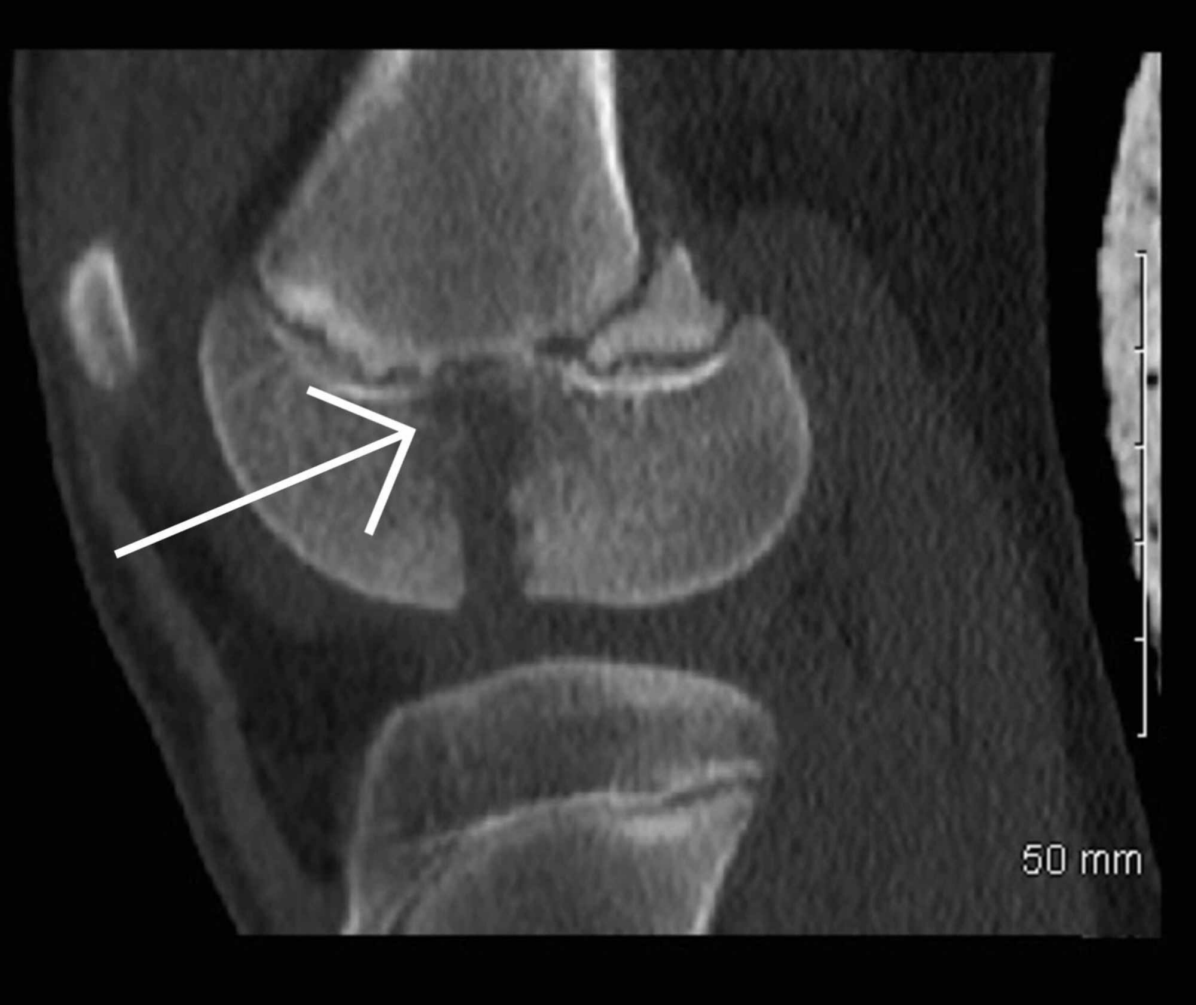
Sagittal view of computed tomography scan of right knee post-injury

**Figure 6:**
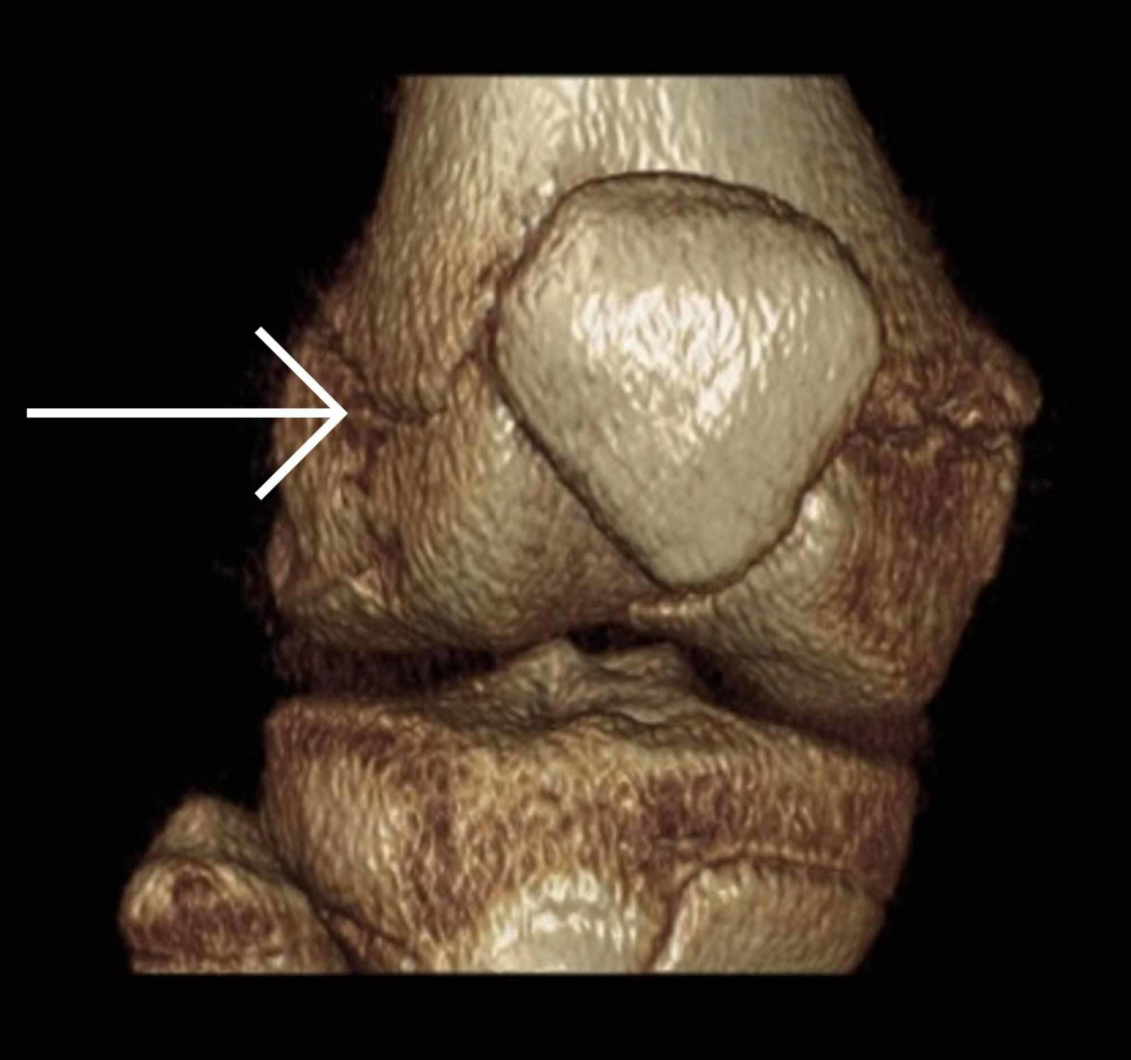
Coronal view of a computed tomography reconstruction post-injury

**Figure 7:**
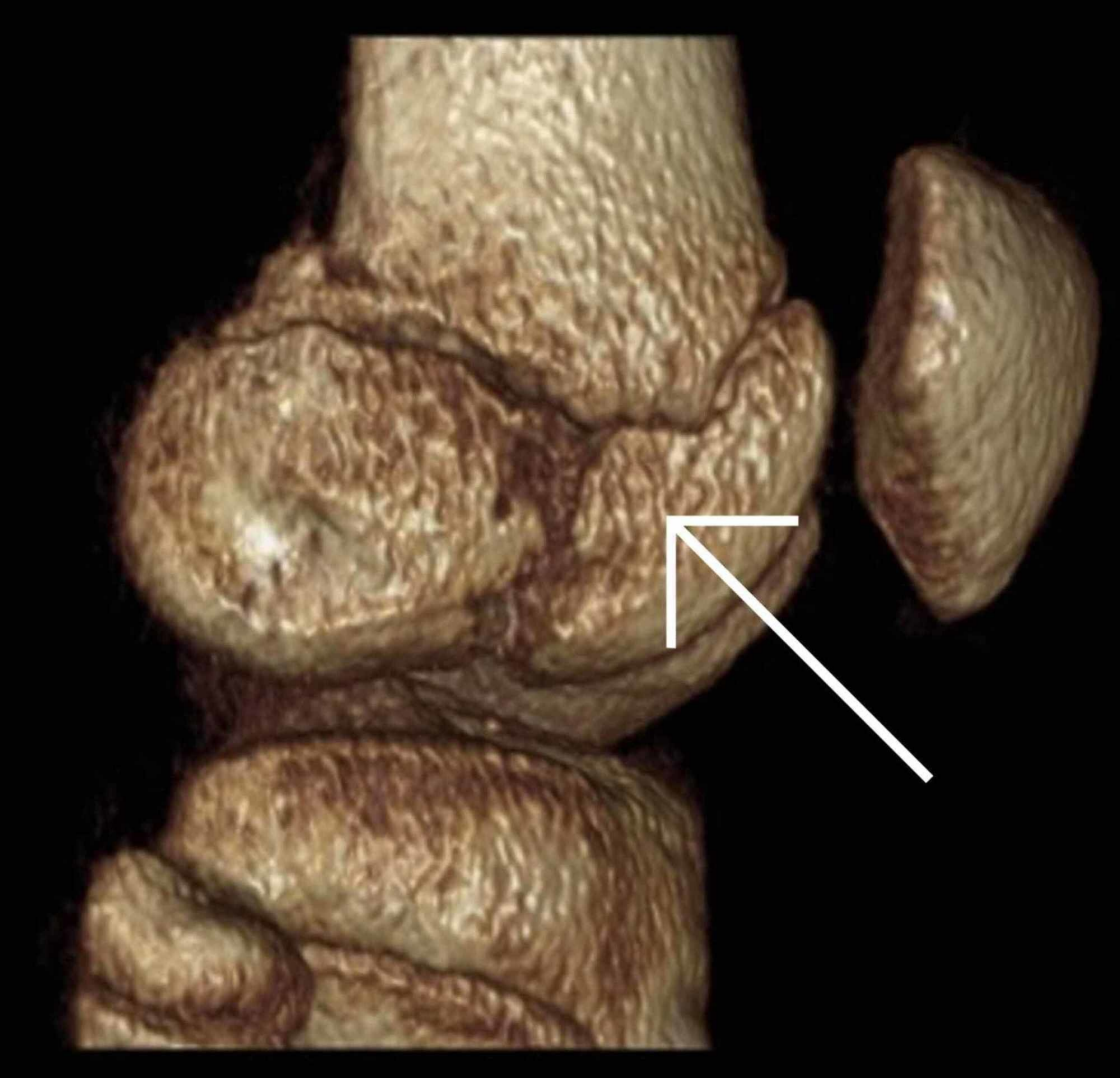
Sagittal view of a computed tomography reconstruction post-injury

**Figure 8:**
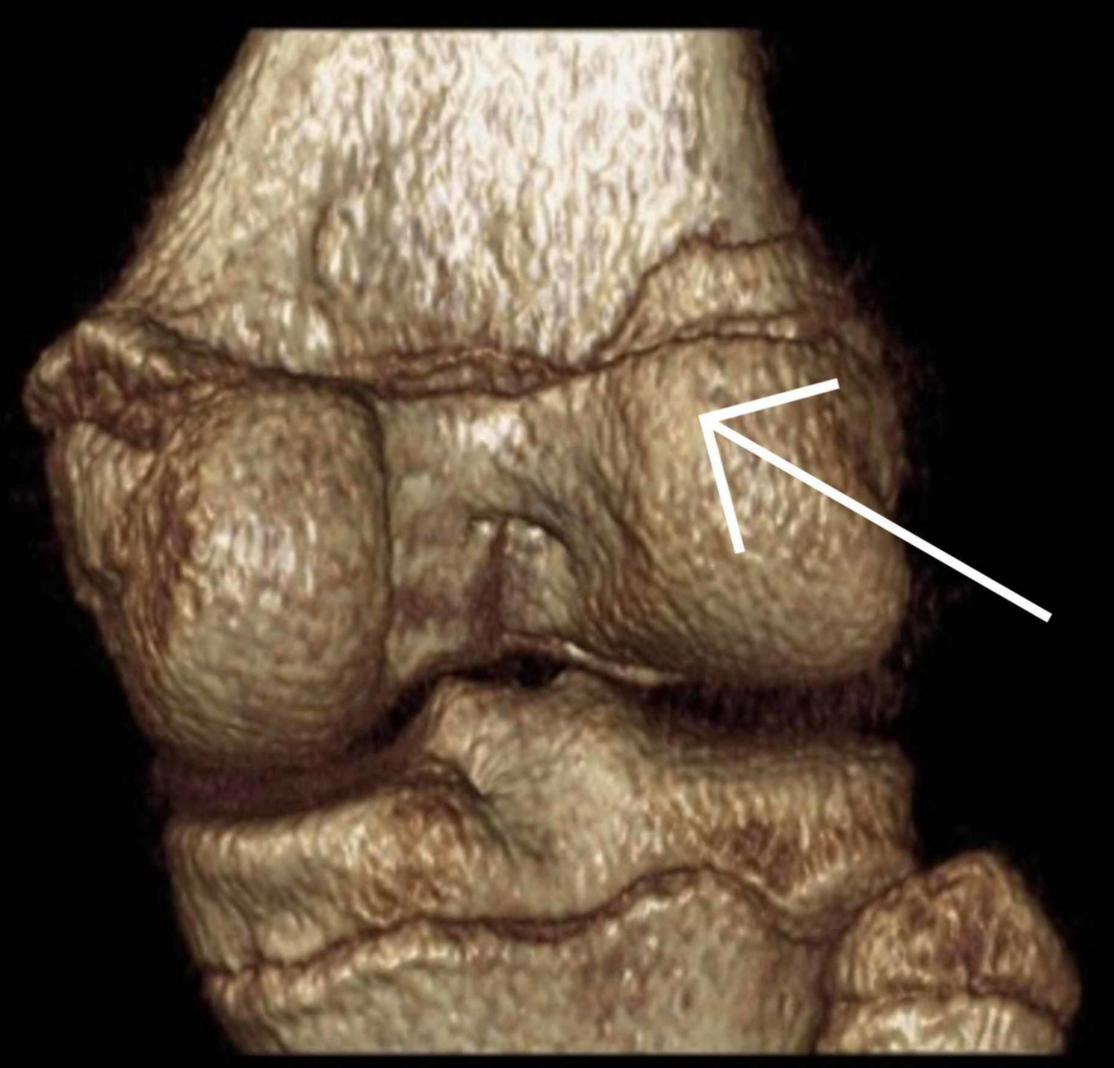
Coronal view of a computed tomography reconstruction post-injury

Once it was established that this was a closed fracture and she was neurovascularly intact, her knee was immobilized in an above knee backslab and transferred to our institution immediately. Upon arrival, she was re-assessed and underwent a CT scan (Figures 3-8) for a definitive diagnosis of her right distal femur fracture. This showed a triplanar fracture of her distal femur. 

**Figure 9:**
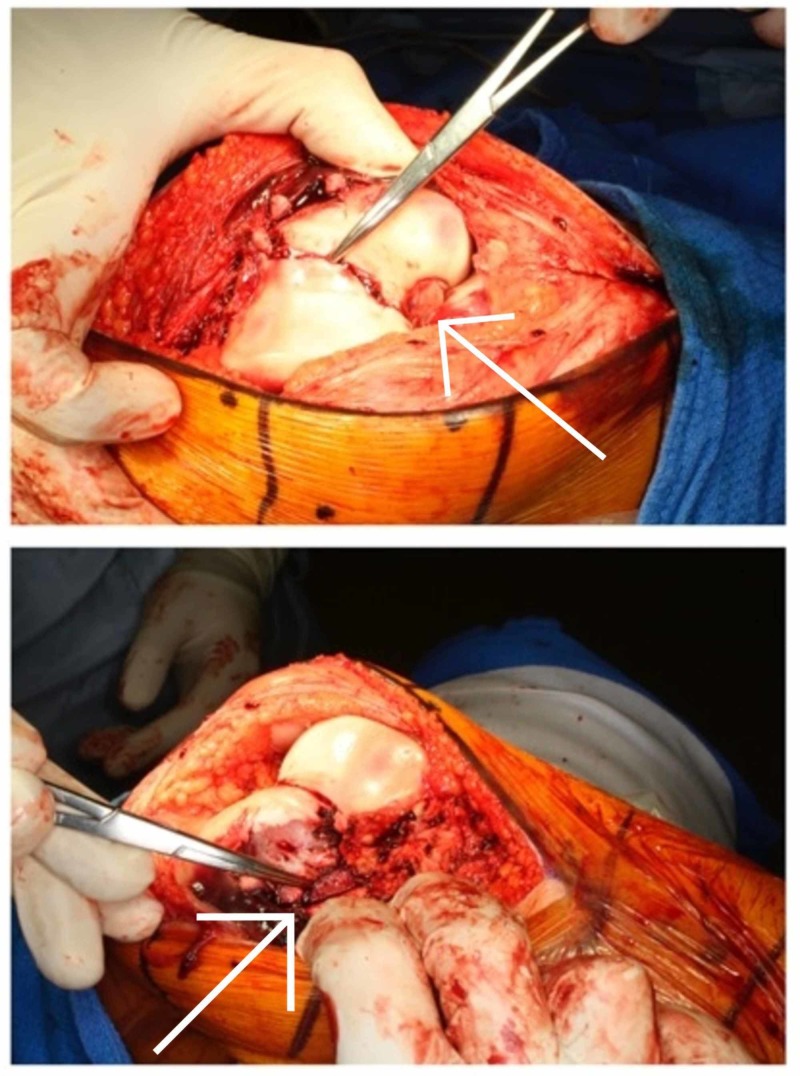
Intraoperative image of right knee

**Figure 10:**
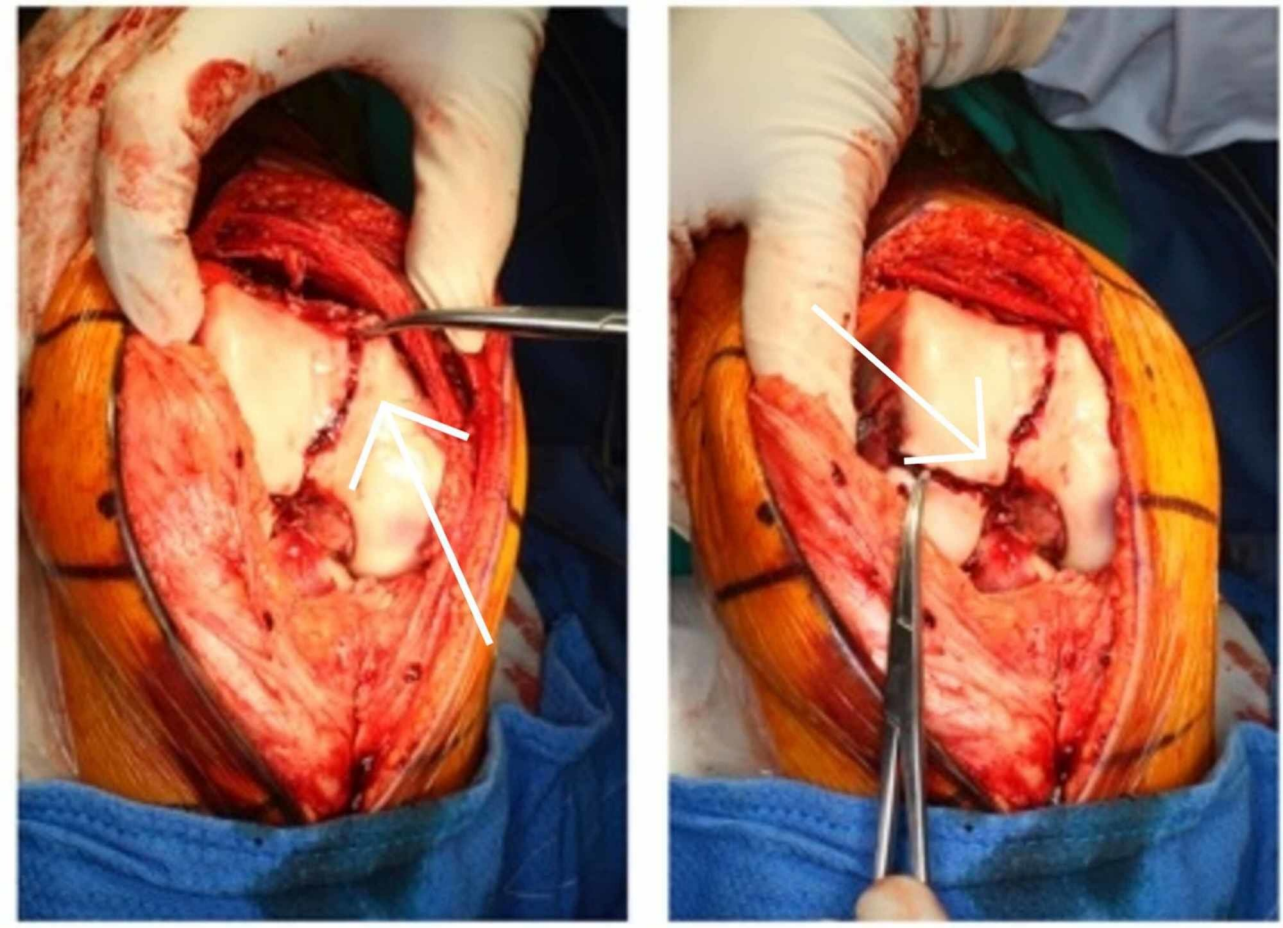
Intraoperative image of right knee

**Figure 11:**
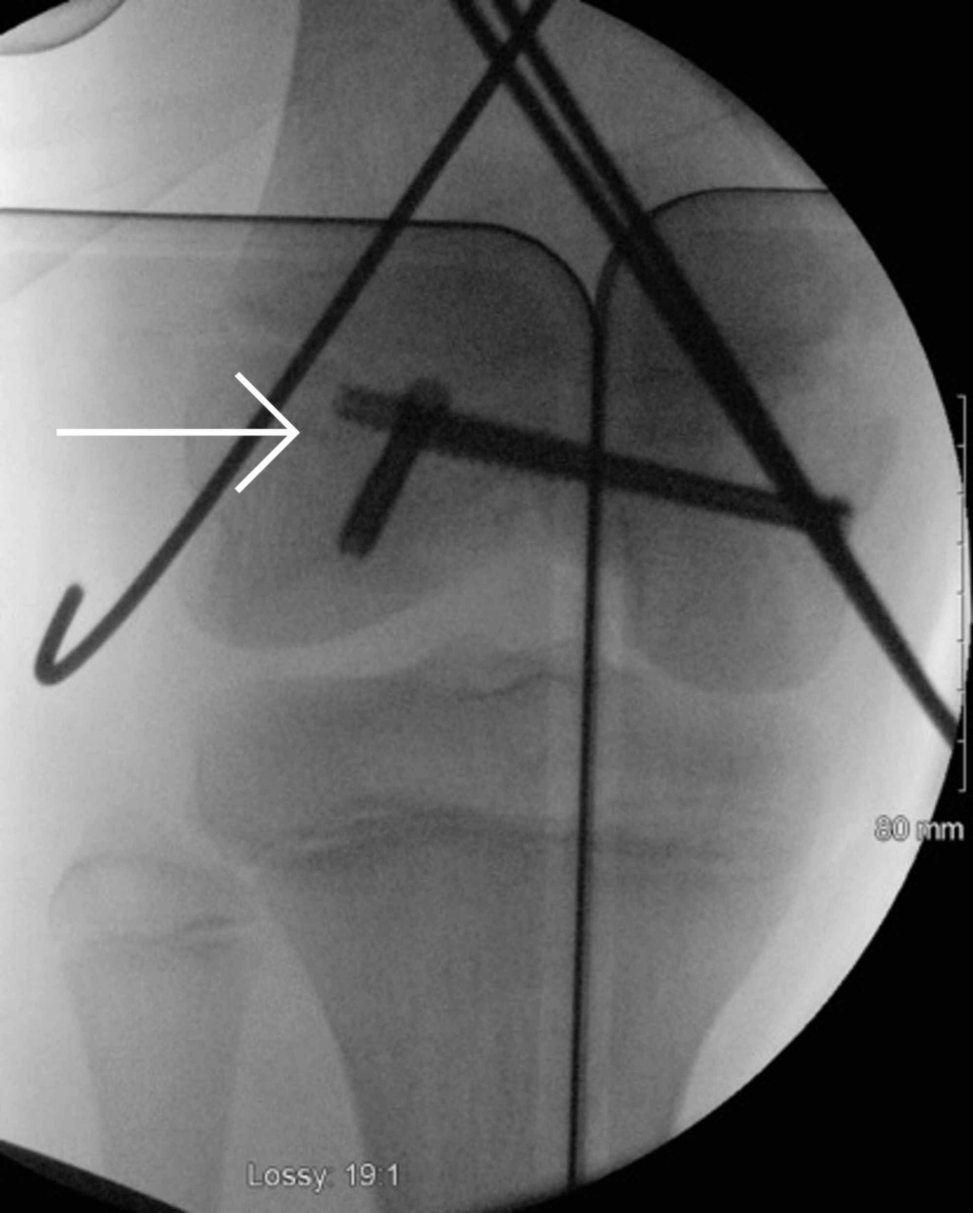
Intraoperative fluoroscopic image of anteroposterior view right knee

**Figure 12:**
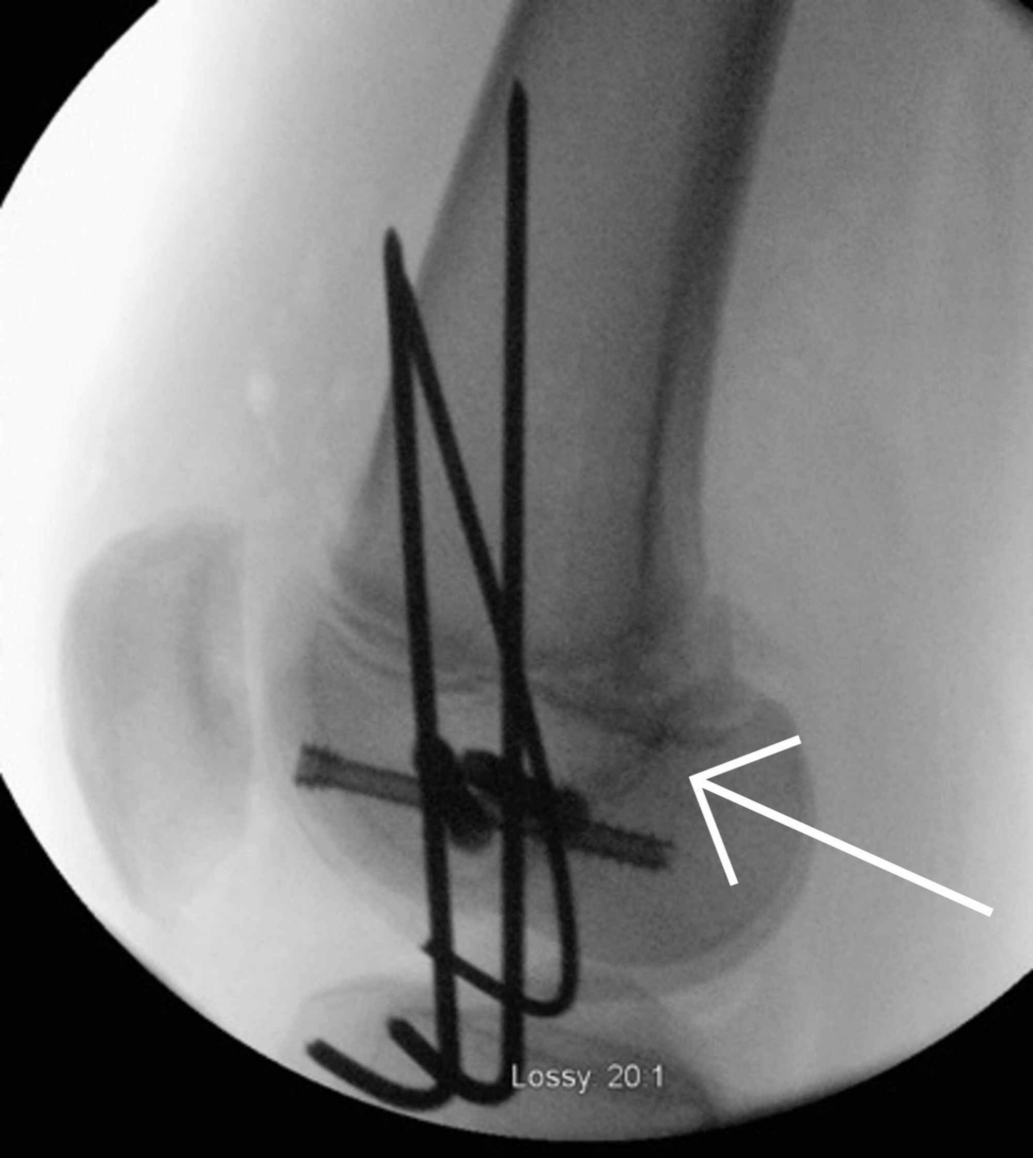
Intraoperative fluoroscopic image of sagittal view right knee

In order to achieve the best possible functional outcome, the trauma and orthopaedic surgery team operated with the goal of anatomical reduction (Figures 9-12). 

Operative technique

**Figure 13:**
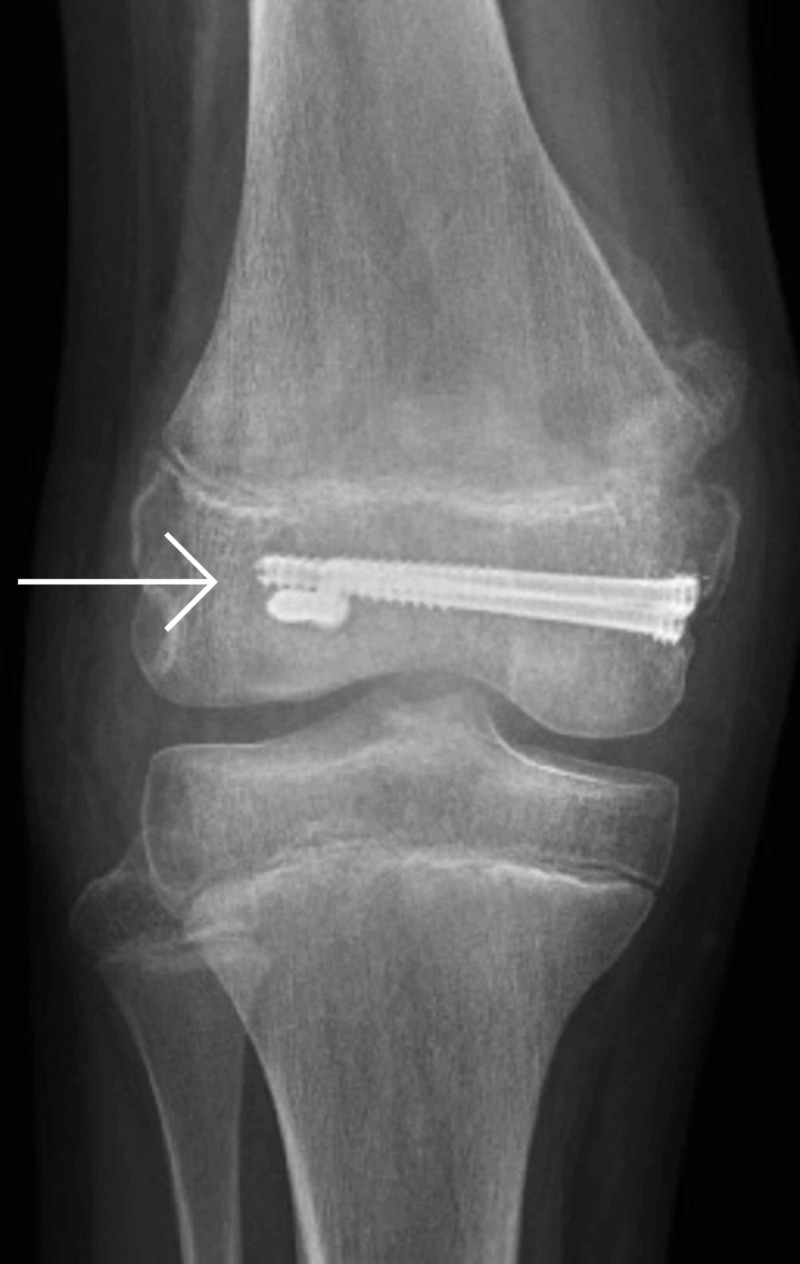
Post-operative anteroposterior x-ray post Kirschner wire removal

**Figure 14:**
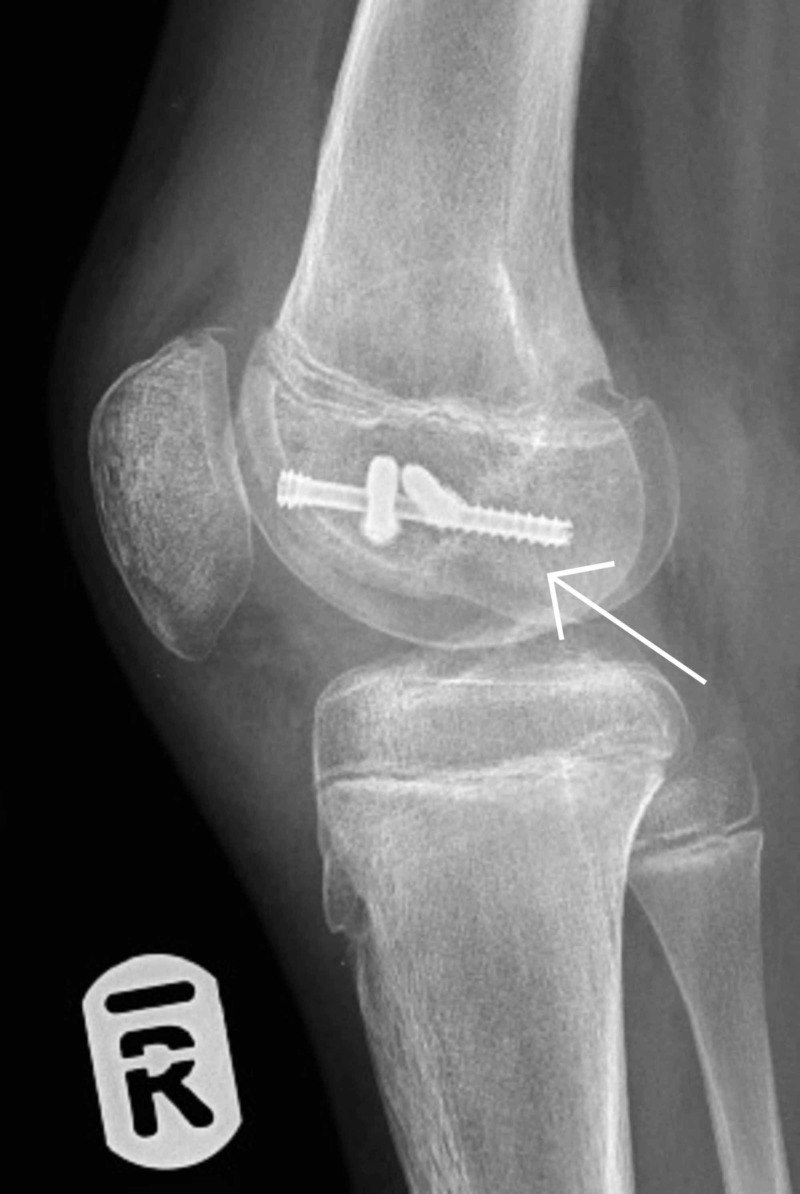
Post-operative lateral x-ray post Kirschner wire removal

An open medial parapatellar approach to the knee was performed. The anatomical reduction was achieved using three cannulated compression screws inserted under fluoroscopic guidance. Step 1: the Hoffa type lateral condylar fracture was reduced and fixed with an anteroposterior headless compression screw [16]. This was counter-sunk. Step 2: the medial condylar fragment was reduced and fixed with two headless compression screws. Anatomic reduction of the articular surface was performed. Medial physis comminution was present. Step 3: two medial 2.5mm Kirschner wires and one 2.5mm lateral Kirschner inserted to capture the posterolateral fragment. These wires were subsequently removed four weeks later (Figures 13 and 14).

Post-operative course

The patient was immobilized in a knee extension brace and kept non-weight bearing for four weeks post-operatively until the wires were removed. Once removed, weight-bearing and physio were commenced. The patient recovered from the injury without any initial post-operative complications.

**Figure 15:**
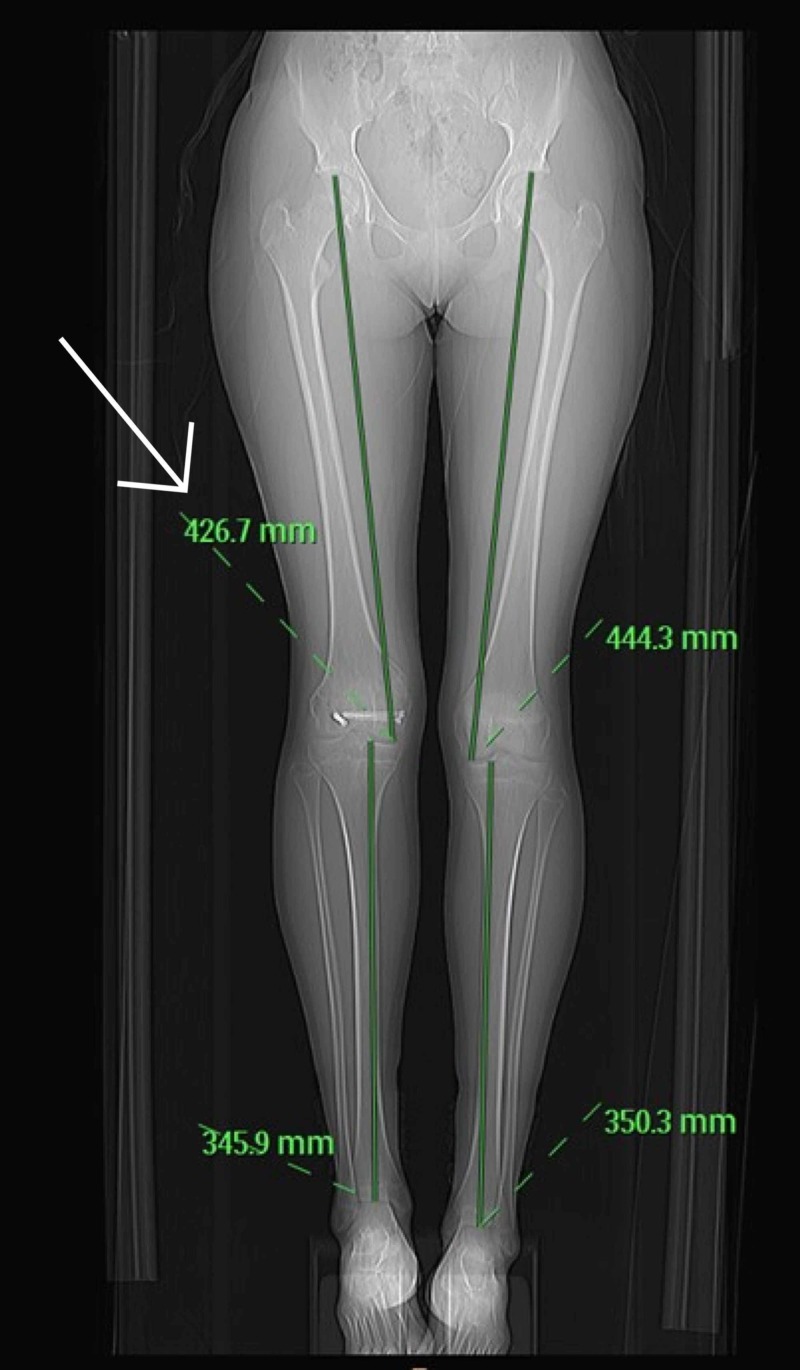
Computed tomography scannogram performed which demonstrated a limb length discrepancy

Limb length discrepancy was noted in October 2017, one-year post-injury. A straight line graph was performed in October 2017. There was a discrepancy of 1.8 cm on the right side. The right leg measured 76.7 cm and left leg 78.5 cm. In January 2018, the limb length discrepancy was 2.2 cm. The right leg was 77.2 cm, and the left leg 79.4 cm (Figure 15).

Proximal tibia epiphysiodesis

**Figure 16:**
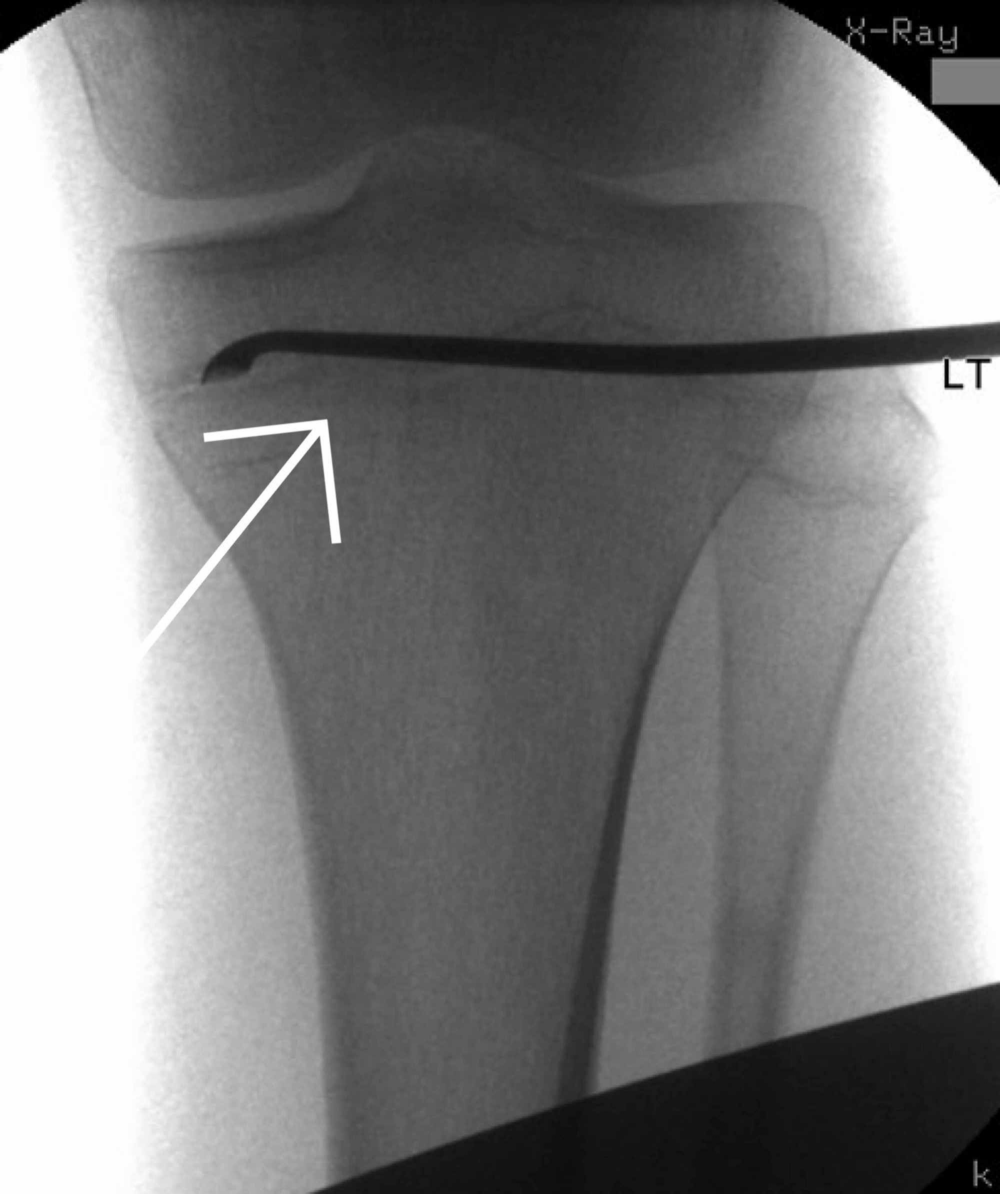
Intraoperative imaging of left proximal tibia epiphysiodesis

In March 2018, a left proximal tibia epiphysiodesis was performed due to the leg length discrepancy (Figure 16). An epiphysiodesis is used to attempt to stop any further growth from occurring at a growth plate by resecting the physical bar [17]. We performed this using a single incision technique and drilling multiple times in the anteroposterior direction across the growth plate of the proximal tibia. A curette was used to ensure the growth plate was completely removed. 

## Discussion

Triplane fractures of the distal femur in the paediatric population are rare, and the majority of trauma and orthopaedic surgeons are not expected to come across this injury throughout their careers. However, in the event that the surgeon does, he or she needs to be able to manage it adequately or else know when to refer. 

Pre-operatively the surgeon must anticipate potential complications and address these with parents. A CT is recommended to plan surgery, and subsequent follow up is crucial to provide the child with the best possible outcome. Regarding the operative technique, it is essential to aim for as much anatomical reduction as possible.

The first documented case of a triplanar distal femur fracture in a child was by Gosselin [6]. This case was of a nine-year-old male who fell from a mango tree and presented to the hospital four to five weeks post-injury. His knee was in 85 degrees of flexion, and there were only a few degrees of active range of motion. The triplanar fracture was confirmed in the operating theatre, and a non-anatomical reduction was achieved and internally fixed with screws. Afterward, the knee had a passive arc of 10 to 90 degrees of motion. Residual growth potential was expected to be nil, and future problems such as pain, angular deformity, and/or leg length discrepancy were anticipated. 

The second documented case was by Masquijo [15]. A 13-year-old boy presented after involvement in a motorcycle accident. He had a CT scan, which confirmed a triplanar fracture. He had a Salter-Harris type I fracture in the axial plane and a Salter-Harris type III fracture in the coronal and sagittal planes of the distal femur. An open procedure was performed with anatomical reduction. This was achieved in a stepwise manner restoring articular congruity and stabilizing the epiphyseal fragment before securing the epiphysis to the metaphyseal component. The first step was to reduce the coronal plane fracture, then secondly, the intercondylar fracture, and thirdly, the reduction of the epiphysis to the metaphysis. At two years of post-operative follow-up, this patient had a knee range of motion from 0-100 degrees. Scanogram revealed a 0.8 cm leg-length discrepancy. He actively participates in bicycle riding and swimming and resumes the same pre-injury activity level. 

The third case is of an eight-year-old girl who was involved in a road traffic accident [4]. She presented with a flexed and painful right knee. Plain film x-ray identified a ‘chondro-epiphyseal fracture of the distal femur dislocated on the coronal plane of the lateral condyle of the right knee’. She was operated on overnight, and a triplanar fracture was identified intraoperatively. An anterolateral approach was performed with medial dislocation of the patella. The fracture was anatomically reduced, and two cannulated screws (5.5 mm) were used. The Hoffa fracture was deemed stable and not fixed. Post-operative follow up at seven years showed good radiographic outcomes (she had removal of the screws at another institution), and clinical examination revealed stability of the right knee with a complete and painless range of motion (-10 to 120 degrees).

## Conclusions

Our case is an example of a rare triplaner femur fracture. We present pre-, peri- and post-operative images of the management of this rare injury. It is imperative to achieve anatomical reduction. CT scanning is recommended to diagnose and manage this injury appropriately. Post-operative follow up is crucial to managing this patient as a limb length discrepancy and/or a deformity is expected.

## References

[1] Hutchinson J (1894). Lectures on injuries to the epiphyses and their results: delivered at the royal college of surgeons. Br Med J.

[2] Peterson CA, Peterson HA (1972). Analysis of the incidence of injuries to the epiphyseal growth plate. J Trauma.

[3] Conroy J, Cohen A, Smith RM, Matthews S (2000). Triplane fracture of the proximal tibia. Injury.

[4] Spagnolo R, Luceri F, Sala F (2016). Pediatric triplane fracture of the distal femur - case report and review of the literature. J Minim Invasive Orthop.

[5] Sponseller PD, Stanitski CL (2001). Distal femoral epiphyseal fractures. Rockwood and Wilkins-Fractures in children.

[6] Gosselin RA, Muteti EN, Beyeza T (2005). A triplane fracture of the distal femoral epiphysis: a case report. East Cent African J Surg.

[7] Tepper KB, Ireland ML (2003). Fracture patterns and treatment in the skeletally immature knee. Instr Course Lect.

[8] Crawford AH (1976). Fractures about the knee in children. Orthop Clin North Am.

[9] Edwards PH Jr, Grana WA (1995). Physeal fractures about the knee. J Am Acad Orthop Surg.

[10] Schnetzler KA, Hoernschemeyer D (2007). The pediatric triplane ankle fracture. J Am Acad Orthop Surg.

[11] Peterson HA (1983). Triplane fracture of the distal humeral epiphysis. J Pediatr Orthop.

[12] Peterson HA (1996). Triplane fracture of the distal radius: case report. J Pediatr Orthop.

[13] Chin KR, Jupiter JB (1999). Treatment of triplane fractures of the head of the proximal phalanx. J Hand Surg Am.

[14] Garcia Mata S, Hidalgo Ovejero A, Martinez Grande M (1999). Triplane fractures in the hand. Am J Orthop.

[15] Masquijo JJ, Allende V (2011). Triplane fracture of the distal femur: a case report. J Pediatr Orthop.

[16] Bartoníček J, Rammelt S. (2015). History of femoral head fracture and coronal fracture of the femoral condyles. Int Orthop.

[17] Ramseier LE, Sukthankar A, Exner GU (2009). Minimal invasive epiphysiodesis using a modified “Canale”-technique for correction of angular deformities and limb leg length discrepancies. J Child Orthop.

